# Editorial On “Exosomes, Their Biogenesis and Role in Inter-Cellular Communication, Tumor Microenvironment and Cancer Immunotherapy”

**DOI:** 10.3390/vaccines8030421

**Published:** 2020-07-28

**Authors:** Vita Golubovskaya, Michael I Bukrinsky, Fabio Grizzi

**Affiliations:** 1 Promab Biotechnologies 2600 Hilltop Drive, Richmond, CA 94806, USA; vita.gol@promab.com; 2 School of Medicine and Health Sciences, The George Washington University, Ross Hall 624, 2300 I St NW, Washington, DC 20037, USA; mbukrins@gwu.edu; 3 Department of Immunology and Inflammation, Humanitas Clinical and Research Center, Via Manzoni 56, 20089 Rozzano, Italy

The term “Exosomes” defines small extracellular vesicles, ranging from 30 to 150 nm in diameter, secreted by most eukaryotic cells into surrounding body fluids including blood, saliva, urine, bile and breast milk. Extracellular vesicles, which used to be considered a garbage disposal pathway, are getting increasing attention from biomedical investigators, and reports demonstrating the role of these factors in multiple physiological and pathological events keep accumulating. Exosomes were first discovered in reticulocytes from sheep in 1983 by two independent groups [1,2]. Exosomes are produced by virtually all cells and can carry numerous cell-derived proteins, DNA, RNA, miRNA [3]. These biologically active molecules are protected by the exosomal membrane from degradative enzymes in the blood, allowing delivery of these molecules to cells located far away from the exosome-producing cell. The exosomal membrane is a lipid bilayer containing proteins, cholesterol, phosphatidylserine, ceramide, sphingolipids, and lipid rafts. The proteins identified in exosomes are mainly involved in multivesicular body formation, membrane transport and fusion (e.g., TSG-101, ALIX), adhesion, and antigen presentation (i.e., MHC class I and II molecules). Additionally, tetraspanins, including CD9, CD63, and CD81, heat shock proteins, principally HSP70 and HSP90, and lipid metabolism-related proteins are often present in exosomes (Figure 1). Exosomes also carry a range of host cell-derived metabolites, DNA fragments and non-coding RNAs, including miRNAs, long noncoding RNAs (lncRNAs), Y-RNAs, fragments of tRNAs, small nuclear and nucleolar RNAs, and piwi-interacting RNAs, and can contain viral RNAs. The ability of exosomes to fuse with target cells facilitates delivery of the exosomal cargo to distant recipients.

The review by Kishore Kumar Jella et al. “Exosomes, Their Biogenesis and Role in Inter-Cellular Communication, Tumor Microenvironment and Cancer Immunotherapy” highlighted an important role of exosomes in cancer and immunotherapy [4]. It has also discussed the role of these vesicles in cell–cell communication between various cell populations through several proposed mechanisms, such as binding to the surface of recipient cells, fusion of vesicles after their adhesion, receptor mediated endocytosis, and phagocytosis, in a wide range of physiological and pathological processes. The exosomes produced by cancer cells play an important role in cancer-related events such as angiogenesis, metastasis, and regulation of the tumor microenvironment [5]. These vesicles also affect cells of the immune system by inhibiting NK-cells, CD4^+^ and CD8^+^ lymphocyte proliferation, thus blocking the anti-tumor immune cell functions [5]. Tucci et al. [6] have also shown a correlation between high levels of CD28 and programmed death receptor 1 (PD-1) in exosomes and the therapeutic response to anti-CTLA4 immunotherapy in metastatic melanoma. On the other hand, exosomes can be used for anti-cancer immunotherapy by loading them with immunotherapy drugs and immunostimulants and targeting them to cancer cells. Exosomes from dendritic cells pulsed with peptides were able to stimulate and activate T-lymphocytes against tumors [7].

However, cancer is only one of many diseases where exosomes are important pathogenic factors but can also be used therapeutically. Conjugating the exosomes with antibody recognizing the antigen expressed in the tissue affected by the autoimmune disease, and loading them with an immunosuppressive therapeutic agent, may provide an effective treatment for autoimmune diseases, such as rheumatoid arthritis, by delivering the therapy directly to affected tissues. A potential protective role of exosomes has been also reported in progressively debilitating neurodegenerative conditions that lead to motor and cognitive dysfunction, such as Parkinson disease [8]. The same concept may be used for treating infectious diseases, including the current pandemic of COVID-19. Here, using exosomes coated with an antibody to Angiotensin-converting enzyme 2 (ACE2), a receptor to SARS-CoV-2, and loading them with antiviral drug, such as remdesivir, would deliver the drug to cells targeted by the virus, thus greatly reducing the side effects. Exosomes are major players in viral pathogenesis. For example, the effect of Human Immunodeficiency Virus (HIV) infection on cholesterol metabolism, the key event in pathogenesis of several HIV-associated co-morbidities, is mediated to a large extent by exosomes carrying the viral protein Nef [9]. Exosomes released from cells infected with such viruses as HIV, Hepatitis C Virus (HCV), human T-cell lymphotropic virus (HTLV), and Dengue Virus (DENV) harbor and deliver many regulatory factors, including viral RNA and proteins, viral and cellular miRNAs, and other host functional genetic elements to neighboring cells, helping to establish productive infections and modulating anti-viral cellular responses [10]. Therefore, targeting exosomes carrying pathogenic viral factors is another, yet unexplored, therapeutic strategy.

An acknowledged advantage of exosomes is their size in the nano range and the fact that they are shed by both natural and abnormal cells, and therefore can be isolated from the host’s own cells [4]. However, studies of body fluid-derived exosomes still remain a challenge, since the isolated exosomes are derived from many different cell types and contain components of body fluids (“contaminants”), which have to be removed during exosome isolation. The utility of exosomes as disease markers is challenged by multiple factors that can influence exosome production and properties. For example, hypoxia has been reported as an important environmental factor that affects exosome production [11]. The field of exosomal research is rapidly advancing, but recent technological progress in exosome isolation and their analysis has not yet led to a universally accepted recommendation for exosome isolation [12]. Additionally, approaches to data acquisition/analysis vary widely and need unification. 

Exosomes can play an important role in the diagnostics of cancer as a biomarker, in infectious diseases as a target of therapeutic interventions, in vaccine development and anti-viral therapy as a delivery vehicle (Figure 1). Future studies will be important to reveal the detailed molecular mechanisms of the biological and immunological functions of exosomes.

## Figures and Tables

**Figure 1:**
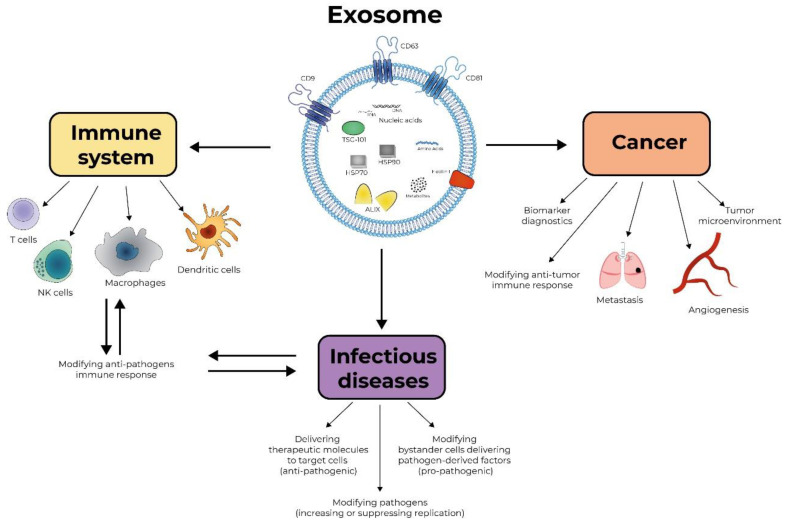
Exosomes as therapeutic and pathological entities. The cartoon depicts the possible involvement of exosomes in pathology and treatment of cancer and the infectious and immunological diseases described in the text.
